# J147 ameliorates sepsis-induced depressive-like behaviors in mice *by* attenuating neuroinflammation through regulating the TLR4/NF-κB signaling pathway

**DOI:** 10.1007/s10735-023-10147-4

**Published:** 2023-09-07

**Authors:** Fang Qiu, Changchun Zeng, Yuqiang Liu, Haobo Pan, Changneng Ke

**Affiliations:** 1 Department of Burn and Plastic Surgery, Shenzhen Longhua District Central Hospital, Shenzhen, 518110 Guangdong China; 2 Center for Human Tissues and Organs Degeneration, Shenzhen Institutes of Advanced Technology, Chinese Academy of Sciences, Shenzhen, 518055 Guangdong China; 3 Department of Medical Laboratory, Shenzhen Longhua District Central Hospital, Shenzhen, Guangdong China; 4 Department of Anesthesiology, Shenzhen Second People’s Hospital, The First Affiliated Hospital of Shenzhen University, Shenzhen, 518025 Guangdong China

**Keywords:** J147, Microglia, Inflammation, Depressive-like behaviors, TLR4, NF-κB

## Abstract

Neuroinflammation is associated with the pathophysiology of depression. The molecular mechanism of depressive-like behavior caused by sepsis-associated encephalopathy (SAE) is incompletely understood. J147 (an analog of curcumin) has been reported to improve memory and has neuroprotective activity, but its biological function in the depressive-like behavior observed in SAE is not known. We investigated the effects of J147 on lipopolysaccharide (LPS)-induced neuroinflammatory, depressive-like behaviors, and the toll-like receptor 4 (TLR4)/nuclear factor-κB (NF-κB) signal pathway in the mouse hippocampus and microglia (BV2 cells). The forced-swimming test (FST) and tail-suspension test (TST) were undertaken for assessment of depressive-like behaviors. Expression of the proinflammatory genes interleukin (IL)-6, IL-1β, and tumor necrosis factor (TNF)-α were measured using RT-qPCR and ELISA. Microglia activation was detected using immunofluorescence staining. The TLR4/NF-κB signaling pathway was studied using western blotting and immunofluorescence staining. J147 pretreatment markedly downregulated expression of IL-6, IL-1β, and TNF-α, and the mean fluorescence intensity of ionized calcium-binding adapter protein-1 in microglia. J147 restrained LPS-induced nuclear translocation of nuclear factor-kappa B (NF-κB), inhibitor of nuclear factor kappa B (IκB) degradation, and TLR4 activation in microglia. J147 administration inhibited bodyweight loss, mortality, microglia activation, and depressive-like behaviors in LPS-treated mice. In conclusion, J147 ameliorated the sepsis-induced depressive-like behaviors induced by neuroinflammation through attenuating the TLR4/NF-κB signaling pathway in microglia.

## Introduction

Sepsis has been defined as “life-threatening multiple organ dysfunction caused by a dysregulated host response to infection” (Singer et al. 2016). Sepsis had an estimated mortality of 11 million (10.1–12.0) in 2017, representing 19.7% (18.2–21.4) of all global deaths (Rudd et al. 2020).

Sepsis-associated encephalopathy (SAE) is a type of diffuse dysfunction in the brain, which can range from delirium to coma (Barbosa-Silva et al. 2021; Bourhy et al. 2022). SAE severely reduces the quality of life, prolongs the duration of stay in the emergency department, and increases the risk of in-hospital mortality (Hajj et al. 2018). Thus, sepsis must be prevented and the host immune system strengthened.

Depression is an increasingly common psychiatric disorder in SAE as well as a major social problem that carries a high economic burden in society. According to the World Health Organization, ~ 264 million people worldwide suffer from depression (Lorigooini et al. 2021). However, depression is poorly understood and treated inadequately**.**

Sepsis-induced neuroinflammation in the central nervous system (CNS) is thought to be a potential mechanism of SAE (Meneses et al. 2019). The hippocampus is the critical region in the brain responsible for the regulating emotion (Richardson et al. 2004). Hippocampus-related dysfunction is correlated with depression (MacQueen and Frodl 2011; Roddy et al. 2019). Although the mechanism of depression is not clear, increasing evidence suggests that inflammation is an important pathologic feature of depression (Jia et al. 2021; Wang et al. 2022).

Microglia are immune cells of the CNS and have a vital role in brain functions (Colonna and Butovsky 2017). Microglia in the hippocampus might be linked to the pathogenesis of depression (Xiao et al. 2021a). In the normal, homeostatic CNS, microglia exhibit a quiescent state. If microglia are activated rapidly following infection or insult, they can release cytotoxic molecules such as proinflammatory cytokines (e.g., interleukin (IL)-6, IL-1β, tumor necrosis factor (TNF)-α), reactive oxygen species (ROS) and proteinases to induce neurotoxicity and neuronal apoptosis (Dheen et al. 2007). Microglia activation is thought to be a critical determinant of neurodegenerative diseases, including Parkinson’s disease, Alzheimer’s disease, and multiple sclerosis (Hickman et al. 2018). Therefore, modulation of microglia activity seems to be a new efficacious therapeutic strategy for depression.

Lipopolysaccharide (LPS) is a component of the outer membrane of Gram-negative bacteria, and induces inflammation (Qin et al. 2007). The binding of LPS to toll-like receptor (TLR)4 triggers signaling cascades, including nuclear factor-kappa B (NF-κB) and mitogen-activated protein kinase (MAPK), which leads to the release of various inflammatory mediators (Zhang et al. 2021). TLR4/NF-κB signaling pathway is critical to microglia activation and neuroinflammation.

J147 is an analog of curcumin. It was designed and synthesized to have high potency and bioavailability. Also, it has neurotrophic effects. It was developed originally to treat neurodegenerative diseases associated with aging (Chen et al. 2011). J147 can target mitochondrial adenosine triphosphate (ATP) synthase, increase levels of brain-derived neurotrophic factor (BDNF), improve memory, reduce infarct volume in embolic occlusion of the middle cerebral artery, and protect against diabetic mellitus-induced neuropathy (Prior et al. 2013; Daugherty et al. 2018; Goldberg et al. 2018; Li et al. 2020b; Pan et al. 2021; Jin et al. 2022). However, whether J147 modulates LPS-induced microglia activation and neuroinflammation in depression is not known.

We hypothesized that J147 could inhibit the activation of microglia and attenuate inflammation, and ameliorate depressive-like behaviors, thereby playing a part in the treatment of inflammation-related CNS diseases. The present study was designed to investigate the anti-inflammatory effects of J147 in LPS-induced microglia through the TLR4/NF-κB signaling pathway and provide a theoretical basis for J147 to treat depression.

## Materials and methods

### Animals

Experiments involving animals were carried out according to guidelines set by the Animal Care and Use Committee of Guangdong Medical University (GDY2202319; 20 May 2022).

Male wild-type (C57BL/6J) mice (8–10 months; GemPharmatech, Guangdong, China) were used for experimentation. All animals were housed in groups of 3–5 in a standard polycarbonate mouse cage (48.8 × 4.8 × 18 cm) and were provide with food and water available ad libitum. They were housed in an animal room at 23 ± 1 °C, humidity of 40%, and exposed to a 12-h light–dark cycle.

Animals were divided randomly into three groups: (1) control (Ctrl); (2) LPS treatment (LPS); (3) LPS combined with J147 (LPS + J147). Behavioral tests were conducted after LPS treatment (n = 8). After that, mice were killed and hippocampus tissues harvested to measure microglia activation (n = 3 mice/group), mRNA expression of proinflammatory cytokines (n = 4–6 mice/group) and protein expression (n = 3 mice/group).

### Drug administration

J147 was purchased from Selleck Chemicals (catalog number: S5269; Houston, TX, USA) and dissolved in 2% dimethyl sulfoxide and phosphate-buffered saline (PBS: 0.01 M) to make a final concentration of 2.5 μM. Then, J147 (10 mg/kg bodyweight) was administered by oral gavage to mice for five consecutive days (Lian et al. 2018; Jin et al. 2022). Mice in the control group were administered 0.01 M PBS. Mouse BV2 microglial cell line was obtained from Shenzhen University, and were pretreated with J147 (2.5 μM) for 2 h before LPS treatment. This concentration of J147 was selected based on cell-viability assay.

### LPS treatment and samples harvesting

LPS treatment was used to activate the TLR4/NF-κB signaling pathway (Yang et al. 2022). Fresh LPS (L2630; MilliporeSigma, Burlington, MA, USA) was dissolved 0.01 M PBS.

For in vivo experiments, mice were administered LPS (5 mg/kg bodyweight) (Arioz et al. 2019). Control-group mice received an equal volume of 0.01 M PBS. Body weight was assessed at 1–8 days post-treatment. Behavioral tests were conducted by researchers blinded to the experimental protocol. After behavioral tests had been completed, one batch of mice was killed and the hippocampus harvested immediately. Hippocampal tissues were stored at − 80 °C until assay. The remaining mice were anesthetized with 1.5% sodium pentobarbital (0.09 mg/g bodyweight, i.p.) and then underwent intracardial perfusion with 0.9% saline followed by 4% paraformaldehyde (BL539A; Biosharp, Beijing, China). After cardiac perfusion, samples of brain tissue were dissected out rapidly, fixed in 4% paraformaldehyde for 24 h at 4 °C, and then dehydrated in sucrose solution (10%, 20%, or 30%). After that, brain samples were embedded in optimal cutting temperature (OCT) compound (ch07274; Sakura Finetek, AV Alphen aan den Rijn, the Netherlands). Coronal sections from hippocampal tissue were cut at a thickness of 25 µm on a cryostat (CM1950; Leica, Wetzlar, Germany).

For in vitro experiments, microglia (BV2 cells) were plated at 5 × 10^4^ cells in six-well plates. Then, they were cultured in Dulbecco’s modified Eagle’s medium (Thermo Fisher Scientific, Waltham, MA, USA) with 2% fetal bovine serum (Thermo Fisher Scientific) plus 1% penicillin and streptomycin (100 U/mL; Thermo Fisher Scientific) in an incubator in an atmosphere of a 5% CO_2_ at 37 °C. BV2 cells were induced with LPS (1 μg/mL) for 24 h. BV2 cells were activated by LPS (1 μg/mL) and divided randomly into four groups: control (Ctrl); J147 (2.5 μM) plus control (Ctrl + J147); LPS (LPS); J147 (2.5 μM) plus LPS (LPS + J147).

### Cell viability assay

The viability of BV2 cells was measured using Cell Counting Kit (CCK)-8 (BS350B; Biosharp) according to manufacturer instructions. BV2 cells (5 × 10^4^) were seeded into in 96-well plates and incubated with J147 for 26 h. Then, CCK-8 solution was added to each well, followed by incubation for the indicated times. Cell viability was calculated by measuring the absorbance at 450 nm.

### Enzyme-linked immunosorbent assay (ELISA)

The supernatant of collected BV2 cells was stored at − 80 °C until processing. Protein expression of IL-6 and TNF-α was measured using ELISA kits (EM0121 and EM0183, respectively; Fine Test, Wuhan, China) according to manufacturer instructions.

### Real-time reverse transcription-quantitative polymerase chain reaction (RT-qPCR)

Hippocampal tissue and BV2 cells were collected, washed with ice-cold 1 × PBS, and stored at − 80 °C until analyses of relative gene expression.

Total RNA from whole hippocampal tissue or BV2 cells was isolated using TRIzol® Reagent (Invitrogen, Carlsbad, CA, USA). Isolated RNA was reverse-transcribed into complimentary-DNA using ReverTra® Ace qPCR RT Master Mix with the gDNA Remove kit (Toyobo, Osaka, Japan). RT-qPCR was undertaken using SYBR® Green Real-time PCR Master Mix (Toyobo). Data were obtained using the 2^*−∆∆CT*^ method. The primer sequences we used (forward and reverse, respectively) were: 5′-TAGTCCTTCCTACCCCAATTTCC-3′ and 5′-TTGGTCCTTAGCCACTCCTTC-3′ for IL-6; 5′-CTTCTCATTCCTGCTTGTGG-3′ and 5′-ATGAGAGGGAGGCCATTTG-3′ for TNF-α; 5′-TTCAGGCAGGCAGTATCACTC- 3′ and 5′-GAAGGTCCACGGGAAAGACAC-3′ for IL-1β; 5′-TTTGCAGCTCCTTCGTTGC-3′ and 5′-CCATTCCCACCATCACACC-3′ for β-actin.

### Western blotting

Protein extracts from BV2 cells or whole hippocampal tissues were obtained. Cells or tissues were lysed with RIPA buffer (Beyotime Institute of Biotechnology, Shanghai, China) containing protease/phosphatase inhibitor tablets (Roche, Basel, Switzerland) and stored at − 80 °C until use. Nuclear protein was prepared using a nuclear extraction kit (Beyotime Institute of Biotechnology) according to manufacturer instructions. A bicinchoninic acid assay kit (Beyotime Institute of Biotechnology) was employed to determine protein concentrations. After sodium dodecyl sulfate–polyacrylamide gel electrophoresis, proteins were transferred to polyvinylidene difluoride (PVDF) membranes (Millipore, Bedford, MA, USA). After blockade with 5% nonfat milk, PVDF membranes were incubated with the appropriate primary and secondary antibodies. Then, immunoreactivity was detected by a chemiluminescent reagent mounting medium (H1000-10: General Electric, Boston, MA, USA). Images of western blots were analyzed using ImageJ (US National Institutes of Health, Bethesda, MD, USA). The following primary antibodies were used: β-actin (1:3000 dilution; 4970S; Cell Signaling Technology, Danvers, MA, USA), TLR4 (1:1000; sc-293072; Santa Cruz Biotechnology, Santa Cruz, CA, USA), NF-κB p65 (1:1000; 8242S, Cell Signaling Technology), phosphorylated (p)-NF-κB, p-p65 (1:1000; 3033S; Cell Signaling Technology), nuclear factor of kappa light polypeptide gene enhancer in B-cells inhibitor, alpha (IκBα; 1:1000; 4814S; Cell Signaling Technology), p-IκBα (1:1000; 2859S; Cell Signaling Technology), and H3 (1:1000; 4499S; Cell Signaling Technology).

### Immunofluorescence staining

Staining of hippocampal sections involved washing with 1 × PBS and blockade with 5% donkey serum and 0.5% Triton X-100 in PBS for 1.5 h. Then, sections were incubated with primary antibody overnight at 4 °C: anti-ionized calcium-binding adapter protein (Iba1; 1:500; goat polyclonal antibody; ab5076; Abcam, Cambridge, UK). The next day, hippocampal sections were washed thrice with 1 × PBS and incubated with secondary antibody for 2 h at room temperature: Alexa fluor dye-conjugated IgG secondary antibody (1:500; A-21432; Thermo Fisher Scientific).

BV2 cells were fixed with 4% paraformaldehyde for 30 min. After washing and blockade, samples were incubated with primary antibody at 4 °C overnight: anti-Iba1 (1:500; goat polyclonal antibody; ab5076; Abcam) and NF-κB (1:500; 8242S; Cell Signaling Technology). The next day, cells were washed thrice with PBS and incubated with anti-goat Alexa Fluor 488-conjugated (1:500; A-11055; Thermo Fisher Scientific) or anti-rabbit Alexa Fluor 555-conjugated (1:500; A-31572; Thermo Fisher Scientific) secondary antibodies for 2 h at room temperature.

Sections and cell were counterstained with 4′,6-diamidino-2-phenylindole (DAPI, Beyotime Institute of Biotechnology), mounted with Vectashield Antifade mounting medium (Vector). Immunofluorescence images were captured by a confocal laser scanning microscope (LSM 800; Carl Zeiss, Oberkochen, Germany). ImageJ was used for quantitative analyses of images.

### Behavioral tests

The behavioral testing room was lit with 250 lx for all the behavior test, including open field test (OFT), rotarod, forced-swimming test (FST), and tail-suspension test (TST). Mice were given 1 h to adapt to the behavior testing room prior to starting each behavioral test. The background sound in the testing room was masked with 53 dB of white noise throughout all behavioral tests.

### OFT

Locomotor activity behavior were assessed using the OFT (Liu et al. 2023). Mice were placed into the center area of a white plexiglass box (50 × 50 × 50 cm) and allowed to explore freely for 5 min. The total distance traveled (a measure of locomotor activity) were recorded using an overhead digital camera, and digital tracks for each mouse were analyzed by EthoVision XT software (Nodus Information Technology, Wageningen, Netherlands). The chamber was cleaned with 75% ethanol at the end of each test.

Rotarod.

Motor performance was assessed utilizing a rotarod test (Ugo basile® 47650) (Qiu et al. 2020). Mice were placed on an accelerating rotarod and allowed to move freely as the rotation increased from 4 to 40 rpm over 300 s, and the time on the rotatod was recorded. Each animal was allowed to perform 3 trials, with a 15-min rest interval between trials. The mean latency to fall of each mouse was analyzed and used for comparison.

### FST

The experimental animals were subjected to FST at day 7 after the LPS injection. FST test (Qiu et al. 2020; Li et al. 2021) was conducted at room temperature (23–25 °C). Mice were allowed to individually swim in a cylindrical Plexiglass™ container (diameter = 15 cm; height = 30 cm) for 6 min with 24 ± 5 °C water. The entire 6-min session was recorded using a video and analysed later. The duration of immobility in the final 5 min was calculated. Mice were considered be “immobile” if struggling was absent (except for the necessity for keeping the nose above the water). The cumulative duration of immobility was recorded by an investigator blinded to the experimental protocol.

### TST

The experimental animals were subjected to TST at day 6 after the LPS injection. The TST (Lad et al. 2007; Kosari-Nasab et al. 2019) was carried out at room temperature (23–25 °C). Mice were suspended ~ 1 cm from the tip of their tail using medical adhesive tape, ~ 50 cm above the ground. The movement of the animals was recorded by video cameras. Each mouse was tested for 5 min. The duration of immobility in the final 4 min was counted. Mice were considered to be “immobile” if struggling was absent, and periods of passive swaying were also included. The cumulative duration of immobility was recorded by an investigator blinded to the experimental protocol.

### Statistical analyses

Data were analyzed with Prism 7 (GraphPad, La Jolla, CA, USA) using unpaired t-tests with Welch’s correction for comparisons between two groups or one-way ANOVA or two-way ANOVA with repeated measures on the time factor followed by Tukey post hoc analyses when significant interactions were identified, and each experiment was repeated at least three times independently. Sample size selection was made based on the previous reports (Cao et al. 2021; Li et al. 2021). Data are the mean ± SEM. One-way ANOVA followed by Bonferroni post hoc test. *p < 0.05, **p < 0.01, ***p < 0.001, ****p < 0.0001.

## Results

### Effect of J147 on cell viability

**Fig. 1:**
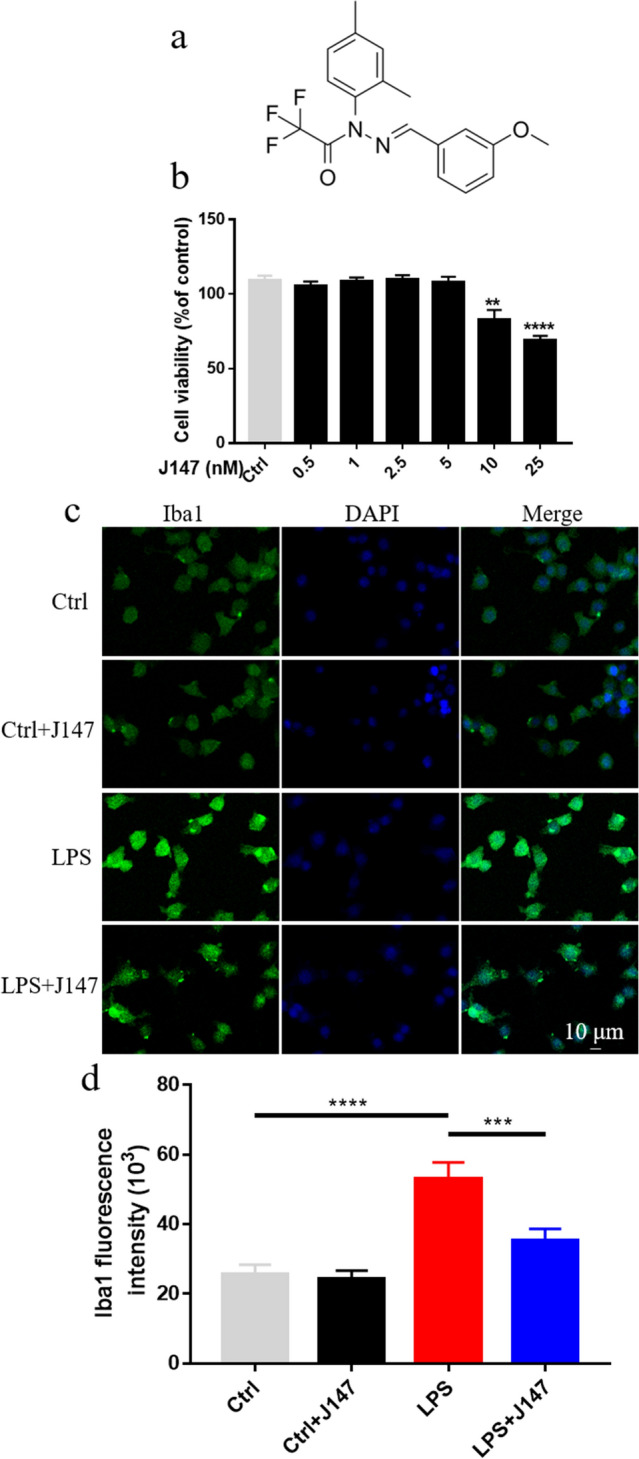
Effects of J147 on the viability of BV2 cells. **a** Chemical structure of J147. **b** BV2 cells were treated with J147 (0 and 25 μM). After 26 h, cell viability was assessed using CCK-8 (n = 8 per group). **c** Activation of BV2 cells was visualized by immunostaining with Iba1 antibody following J147 treatment in the presence of LPS (1 μg/mL) for 26 h. **d** Mean fluorescence intensity of Iba1 (n = 20 per group) (one way ANOVA, *F*_(3_,_76)_ = 15.27, *p* < 0.0001). Data are the mean ± SEM. ANOVA followed by Bonferroni post hoc test. **p < 0.01, ***p < 0.001; ****p < 0.0001

First, we evaluated the viability of BV2 cells after J147 treatment (0–25 μM) for 26 h. J147 at 0–5 μM did not induce cytotoxicity, but J147 > 5 μM did (Fig. 1b). Then, we examined activation of BV2 cells incubated with J147 (2.5 μM) in the presence or absence of LPS (1 μg/mL). The mean fluorescence intensity of LPS-treated BV2 cells was increased compared with that in the control group, which indicated activation of BV2 cells. However, J147 treatment reduced activation of BV2 cells significantly compared with that in the LPS group (Fig. 1c, d).

### J147 inhibited the LPS-induced release of proinflammatory cytokines in BV2 cells

**Fig. 2:**
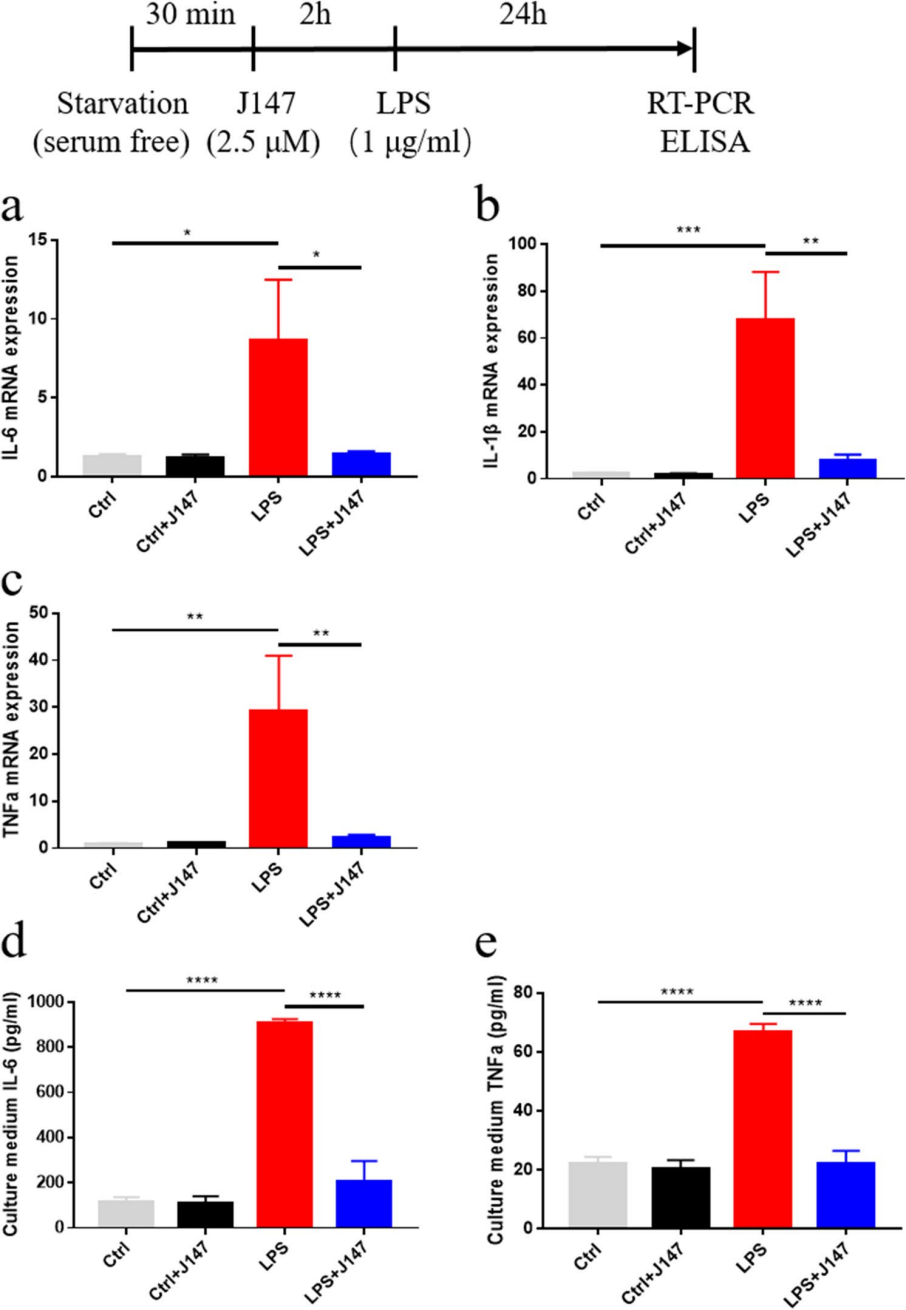
Effect of J147 on expression of proinflammatory cytokines in LPS-induced BV2 cells. BV2 cells were pretreated with J147 (2.5 μM) for 2 h before incubation with LPS (1 μg/mL) for 24 h. **a**–**d** Effect of J147 on mRNA expression of IL-6 (one way ANOVA, *F*_(3_,_20)_ = 3.539, *p* = 0.0333), IL-1β (one way ANOVA, *F*_(3_,_20)_ = 9.469, *p* = 0.0004), and TNF-α (one way ANOVA, *F*_(3_,_20)_ = 5.557, *p* = 0.0061) in BV2 cells. **e** Protein expression of IL-6 (one way ANOVA, *F*_(3_,_20)_ = 59.12, *p* < 0.0001) and TNF-α (one way ANOVA, *F*_(3_,_20)_ = 46.46, *p* < 0.0001) was determined using ELISA kits. Columns present the mean ± SEM (n = 6 per group). ANOVA followed by Bonferroni post hoc test. *p < 0.05, **p < 0.01, ***p < 0.001, ****p < 0.0001

To investigate the potential role of J147, we measured expression of proinflammatory cytokines in LPS-induced BV2 cells using RT-qPCR and ELISA. Compared with the control group, mRNA expression of proinflammatory cytokines (IL-6, IL-1β, TNF-α) was increased markedly in LPS-induced BV2 cells. However, J147 treatment inhibited mRNA expression of proinflammatory cytokines significantly compared with that in the LPS group (Fig. 2a–c). Next, protein expression of proinflammatory cytokines (TNF-α, IL-6) was measured by ELISA. Expression of TNF-α and IL-6 in the culture medium increased after LPS treatment, but J147 treatment inhibited expression of TNF-α and IL-6 after LPS treatment (Fig. 2d, e).

### J147 therapy inhibited the TLR4/NF-κB signaling pathway in LPS-induced BV2 cells

**Fig. 3:**
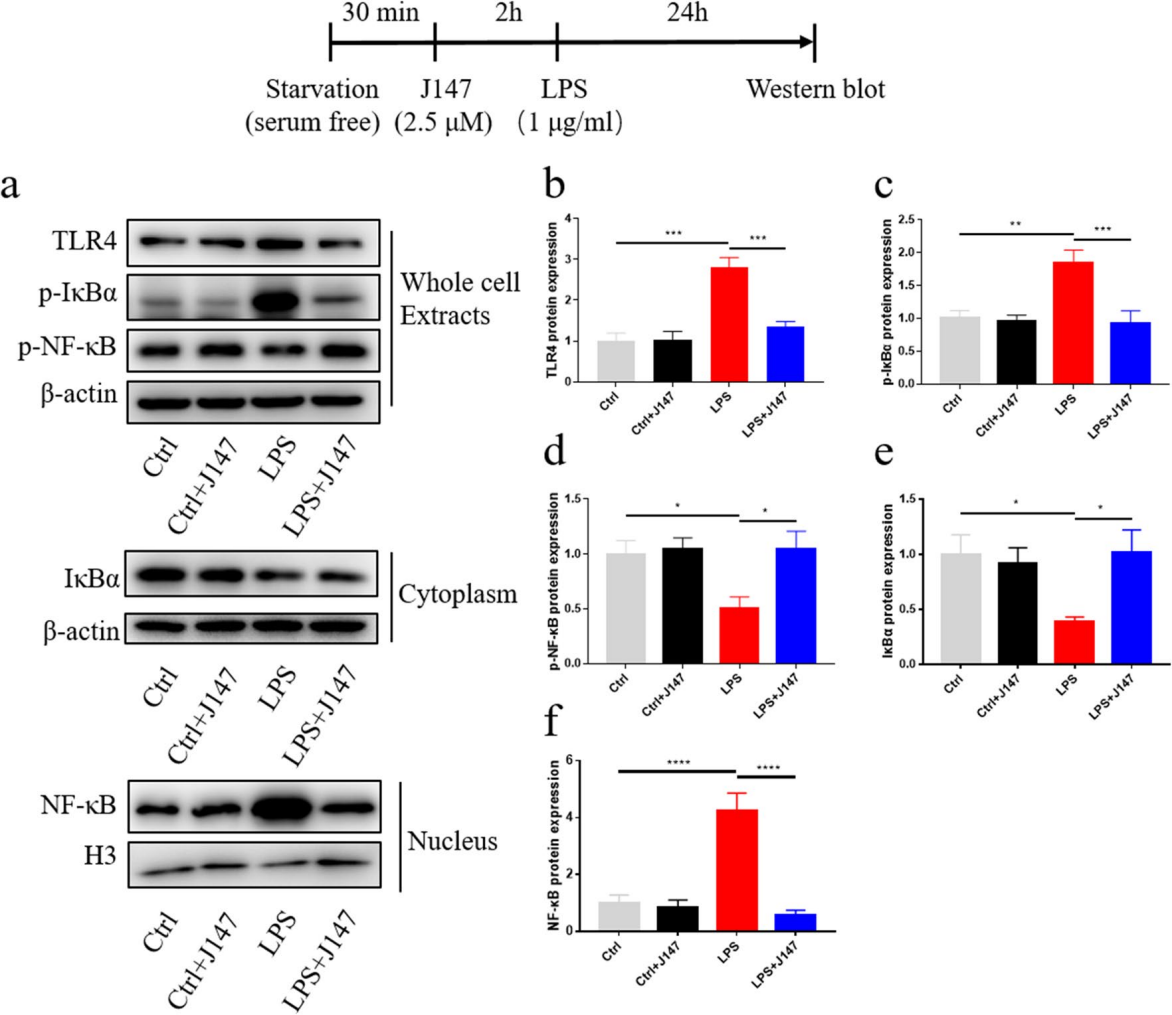
Effect of J147 on the TLR4 and NF-κB signaling pathways in LPS-induced BV2 cells. **a** Protein expression of TLR4 (one way ANOVA, *F*_(3_, _20)_ = 19.59, *p* < 0.0001), p-IκBα (one way ANOVA, *F*_(3_, _20)_ = 8.128, *p* = 0.0010), and p-NF-κB p-p65 (one way ANOVA, *F*_(3_, _20)_ = 4.347, *p* = 0.0164) in whole cells (n = 6 per group), IκBα (one way ANOVA, *F*_(3_, _12)_ = 3.701, *p* = 0.0428) in the cytoplasm and NF-κB p65 (one way ANOVA, *F*_(3_, _12)_ = 21.73, *p* < 0.0001) in the nucleus in J147-treated BV2 microglia cells (n = 4 per group). **b**–**f** Quantitative analysis of the Western blots. Data are the mean ± SEM. ANOVA followed by Bonferroni post hoc test. *p < 0.05, **p < 0.01, ***p < 0.001, ***p < 0.0001

**Fig. 4:**
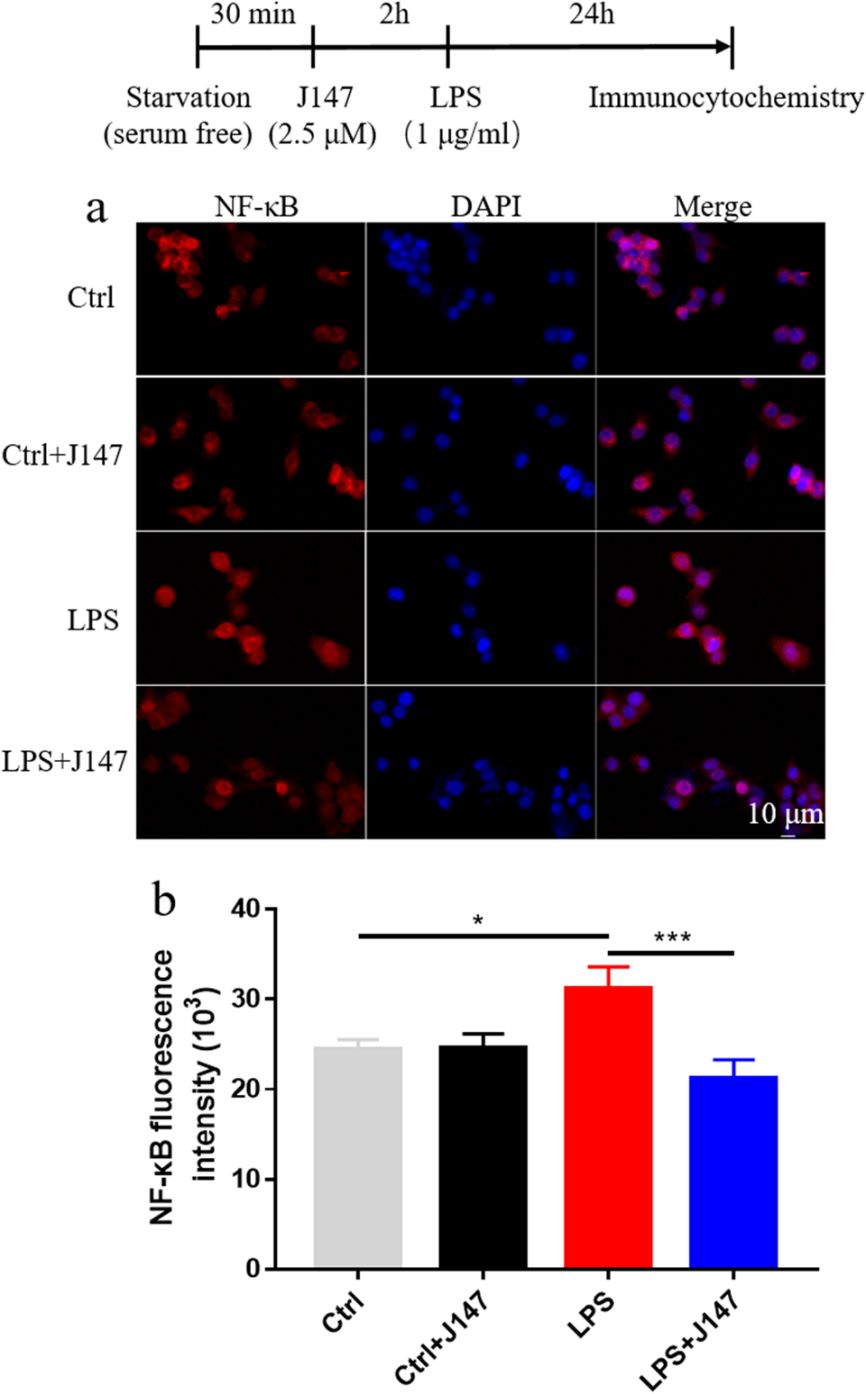
Effect of J147 on the nuclear translocation of NF-κB p65. **a** BV2 cells were pretreated with J147 (2.5 μM) for 2 h before treatment with LPS (1 μg/mL) for 24 h. Localization of NF-κB p65 was visualized by confocal laser scanning microscopy following immunofluorescence staining with an anti-NF-κB antibody (red) combined with DAPI for nuclei (blue). Results are representative of four groups. **b** Quantification of the data shown in **a** (n = 14 per group) (one way ANOVA, *F*_(3_,_52)_ = 4.92, *p* = 0.0044). Data are the mean ± SEM. ANOVA followed by Bonferroni post hoc test. *p < 0.05, ***p < 0.001

The NF-κB pathway has been reported to be closely associated with inflammation (Liu et al. 2019; Yang et al. 2022). Next, we investigated whether J147 treatment inhibited nuclear translocation of NF-κB p65. In whole-cell extracts, protein expression of TLR4 and p-IκBα was increased markedly after LPS stimulation, but p-NF-κB expression was decreased markedly (Fig. 3a–d). The LPS-only group had dramatically reduced expression of IκBα in the cytoplasm and increased expression of NF-κB p65 in the nucleus (Fig. 3a, e–f). Immunofluorescence analyses showed that LPS treatment increased the translocation of NF-κB p65 to the nucleus (Fig. 4). However, these effects were reversed markedly by J147 therapy (Figs. 3, 4).

### J147 treatment alleviated depressive-like behaviors in mice with LPS-induced sepsis

**Fig. 5:**
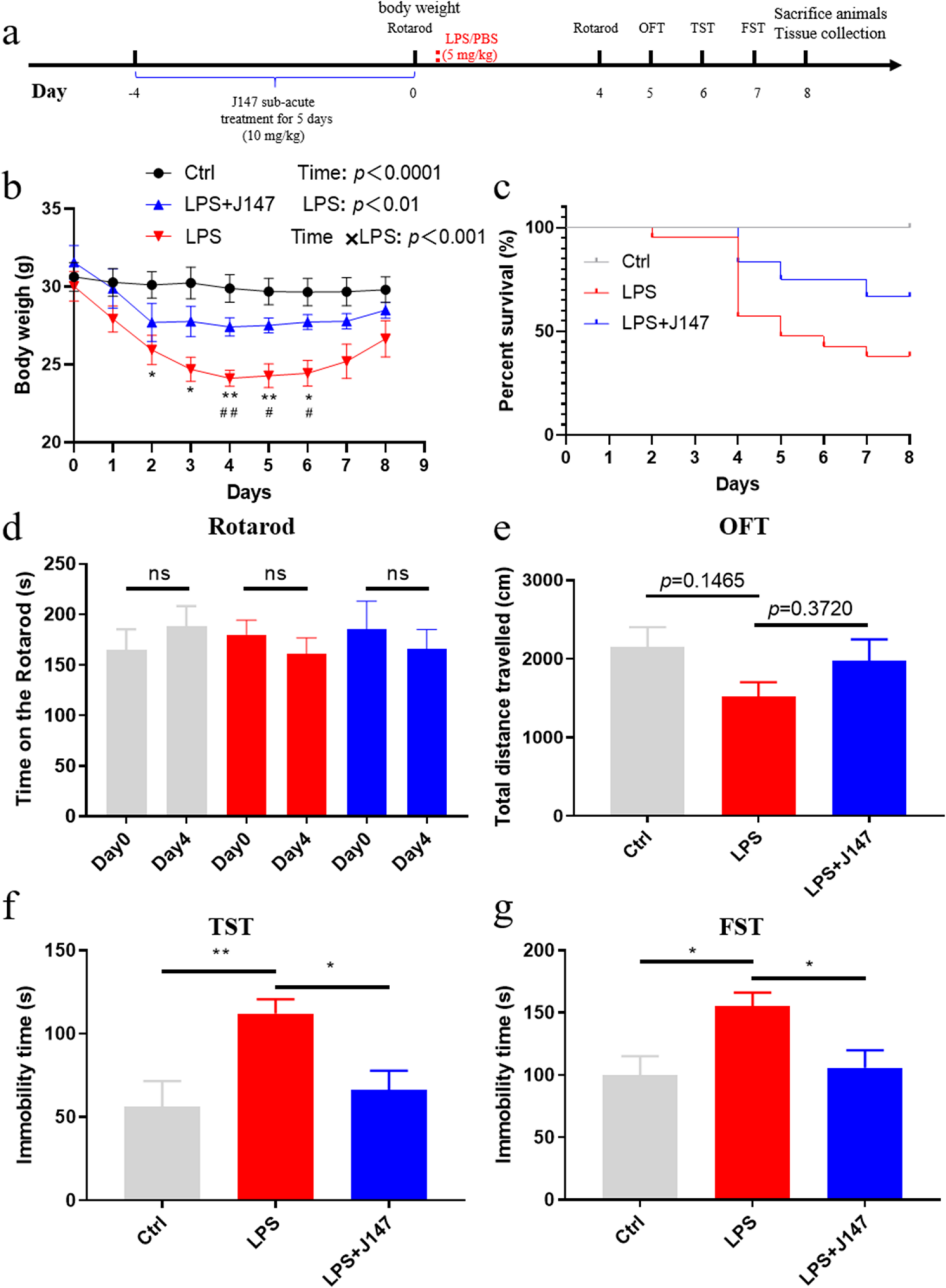
Effects of J147 on depressive-like behaviors in LPS-induced mice. **a** Experimental protocol. **b** J147 treatment prevented the bodyweight loss induced by LPS. Weight loss of mice was expressed as mean ± SEM (n = 8 mice for each group) (two-way ANOVA followed by Turkey post hoc analyses, Time × LPS: F (16, 168) = 3.009, *p* = 0.002; Time: F (1.563, 32.82) = 16.14, *p* < 0.0001; LPS: F (2, 21) = 7.839, *p* = 0.0029), Ctrl vs LPS, *p < 0.05, **p < 0.01; LPS vs J147 + LPS ^#^*p* < 0.05, ^##^*p* < 0.05. **c**. Survival was monitored every day after LPS injection. (Ctrl, n = 8 mice; LPS, n = 21 mice; LPS + J147 mice, n = 12 mice). **d** Performance of rotarod test among three group. (n = 8 mice for each group). (*p* > 0.05). **e** The total distance travelled in OFT test among three groups (one way ANOVA, *F*_(2_,_21)_ = 1.898, *p* = 0.1747). (n = 8 mice for each group). **f** Performance of TST among three groups. (n = 8 mice for each group) (one way ANOVA, *F*_(2_,_21)_ = 5.960, *p* = 0.0089). **g** Performance of FST among three groups. (n = 8 micefor each group) (one way ANOVA, *F*_(2_,_21)_ = 4.985, *p* = 0.0169). Data are the mean ± SEM. ANOVA followed by Bonferroni post hoc test. *p < 0.05, **p < 0.01, ***p < 0.001, ****p < 0.0001

Next, we determined the effects of J147 therapy on behavioral tests in mice with LPS-induced sepsis. The experimental design is shown in Fig. 5a. J147 was administered by daily oral gavage to mice for 5 consecutive days. Then, mice were administered LPS via the intraperitoneal route. After that, mice were monitored daily for body weight and mortality. Our results demonstrated that the body weight was significantly reduced after LPS treatment. However, J147 pretreatment significantly prevented LPS-induced body weight loss (Fig. 5b). Survival was monitored after LPS-treatment for 8 days: J147 therapy significantly reduced LPS-induced death (Fig. 5c). Physical weakness mice were excluded from further behavior tests analysis after LPS-treated. Rotarod test were carried out to assess the motor coordination of mice (Fig. 5d). Before and after LPS treatment at day 0 and 4, mice do not display any significant different performance at the rod. Moreover, OFT was subjected to test locomotor activity (Fig. 5e). There was no significant difference in the total distance among these groups.

Previous studies have reported that LPS was administered to induce depression-like behaviors and neuroinflammation (Ali et al. 2020; Li et al. 2021). TST and FST were carried out to test depressive-like behavior. The results showed that LPS treatment increased the immobility of mice significantly in the TST and FST. Conversely, J147 therapy markedly reduced the immobility of mice in the TST and FST (Fig. 5f, g). Thus, we aimed to explore how J147 therapy rescued depressive-like behaviors in mice with LPS-induced sepsis.

### Effects of J147 therapy on microglia activation in the hippocampus

**Fig. 6:**
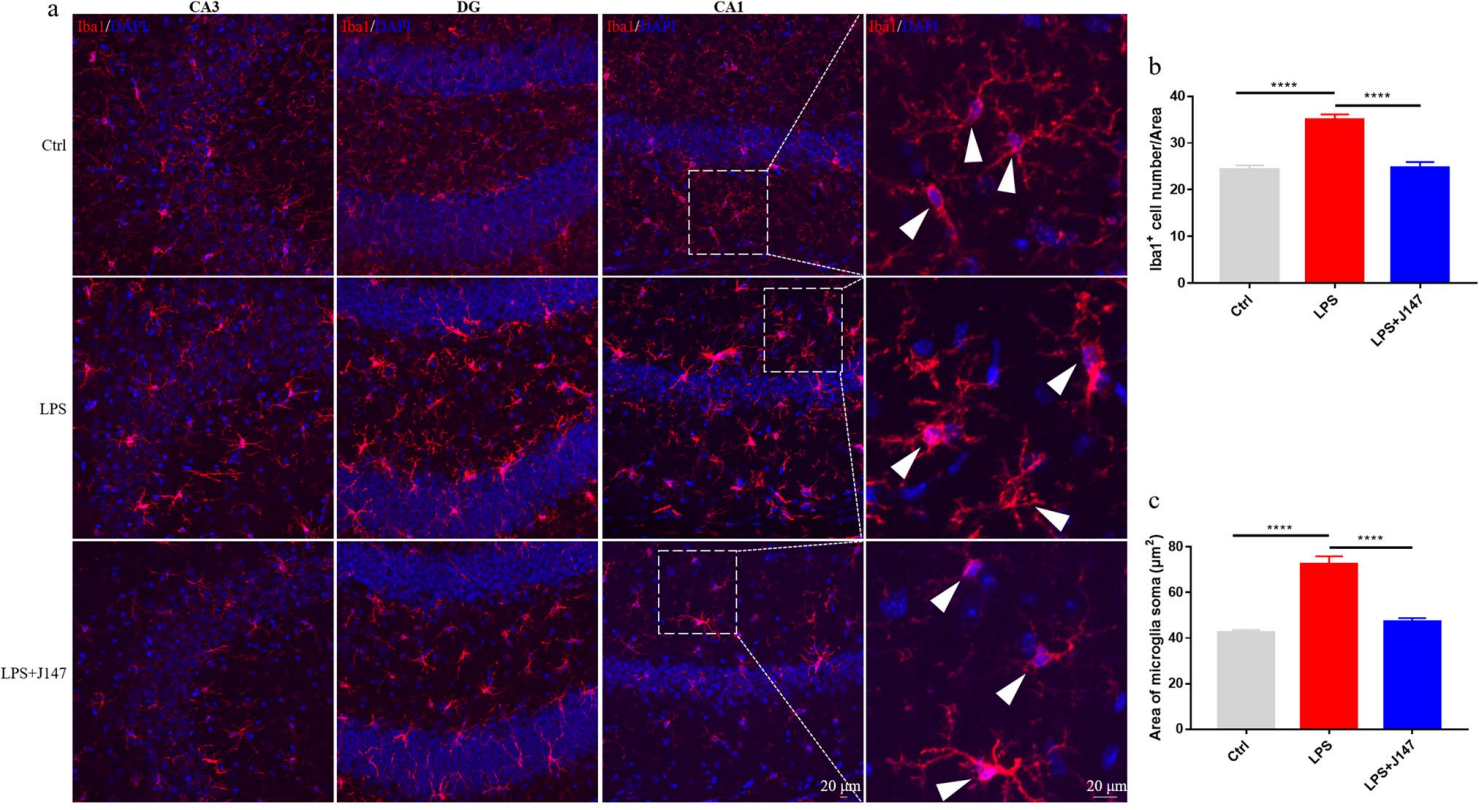
J147 decreased LPS-induced microglia activation in the hippocampus. **a** Immunofluorescence staining of Iba-1 in the CA1 hippocampal area. *Scale bar*, 20 μm. **b** Quantification of Iba1-positive cells in microglia density. (n = 41 brain slice from 3 mice per group) (one way ANOVA, *F*_(2_,_120)_ = 27.3, *p* < 0.0001). **c** Quantification of microglia in the soma. (n = 96 cell from 3 mice per group) (one way ANOVA, *F*_(2_,_285)_ = 50.69, *p* < 0.0001). Data are the mean ± SEM. ANOVA followed by Bonferroni post hoc test. ****p < 0.0001

Microglia have been implicated in the pathogenesis of depression (Brites and Fernandes 2015). To further address if J147 treatment could reduce depressive-like behaviors by inhibiting the inflammatory response, microglia in the hippocampal tissue of mice were labeled with Iba1. LPS exposure significantly enhanced the number and area of microglia compared with that in mice in the control group (Fig. 6). Interestingly, after pretreatment with J147, the significant increase in the number and morphology of microglia in the hippocampus induced by LPS therapy was reversed.

### J147 administration reduced the LPS-induced production of proinflammatory cytokines through TLR4/NF-κB signaling pathway

**Fig. 7:**
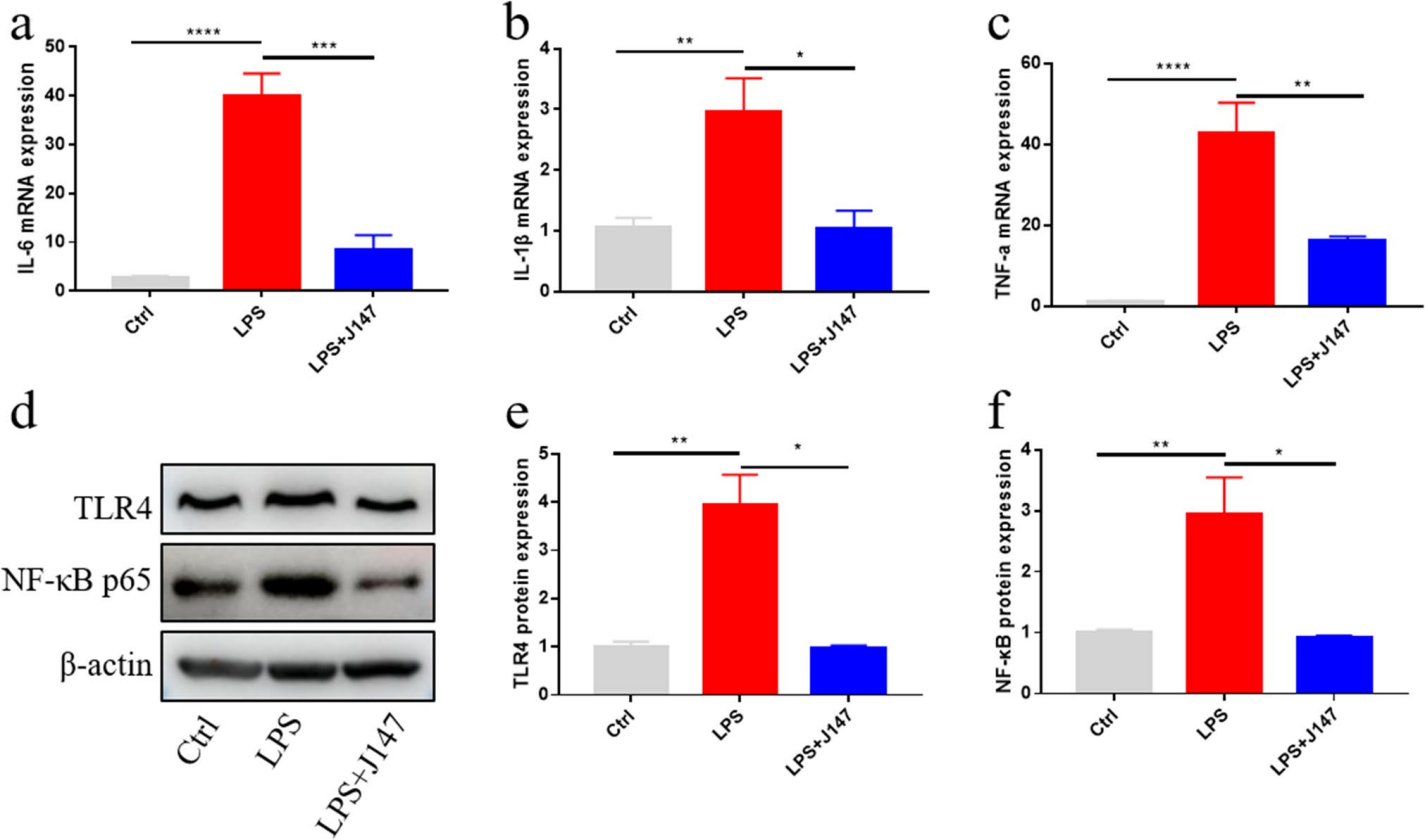
Effect of J147 on LPS-induced neuroinflammatory cytokine levels in mouse hippocampus. **a**–**c** mRNA expression of TNF-a (one way ANOVA, *F*_(2_,_13)_ = 19.88, *p* = 0.0001), IL-1β (one way ANOVA, *F*_(2_, _13)_ = 7.816, *p* = 0.0059) and IL-6 (one way ANOVA, *F*_(2_,_13)_ = 38.69, *p* < 0.0001) measured by RT*-q*PCR (Ctrl, n = 6 mice; LPS, n = 6 mice; LPS + J147, n = 4 mice). **d** Representative Western blot. **e**, **f** Quantitative analysis of **d** in the hippocampus among the three groups (n = 3 mice per group), TLR-4 (one way ANOVA, *F*_(2_,_6)_ = 21.28, *p* = 0.0019), and NF-κB p65 (one way ANOVA, *F*_(2_,_6)_ = 10.97, *p* = 0.0099). Data are the mean ± SEM. ANOVA followed by Bonferroni post hoc test.*p < 0.05, **p < 0.01

Neuroinflammation is involved in SAE pathogenesis, and can lead to anxiety, depression, and cognitive dysfunction (Li et al. 2020a). Based on the findings of the in vitro and in vivo experiments stated above, we explored the mechanism of the anti-inflammatory effect of J147 in vivo. To discover if J147 therapy had effects on inflammation in the hippocampus of mice suffering from sepsis, expression of proinflammatory cytokines (IL-6, IL-1β, TNF-α) in the hippocampus was measured. mRNA expression of proinflammatory cytokines (IL-6, IL-1β, TNF-α) was increased obviously in LPS-induced mice, but J147 pretreatment reduced mRNA expression of proinflammatory cytokines (IL-6, IL-1β, TNF-α) significantly (Fig. 7a–c).

The TLR4/NF-κB signaling pathway has a key role in inflammation (Kim et al. 2017; Liu et al. 2019; Yang et al. 2022). LPS treatment stimulates the TLR4/NF-κB signal, while recruits proinflammatory cytokines and results in cellular injury (Akcay et al. 2009; Zhang et al. 2017). We wished to investigate if J147 therapy had an impact on the TLR4/NF-κB signaling pathway in the hippocampus of LPS-induced mice, so hippocampal expression of TLR4 and NF-κB was measured by western blotting. LPS exposure increased protein expression of TLR4 and NF-κB in the hippocampus significantly, and J147 pretreatment reversed this increase markedly (Fig. 7d–f). In summary, J147 treatment ameliorated neuroinflammation and the depressive-like behaviors of mice suffering from sepsis by inhibiting the TLR4/NF-κB signaling pathway.

## Discussion

**Fig. 8:**
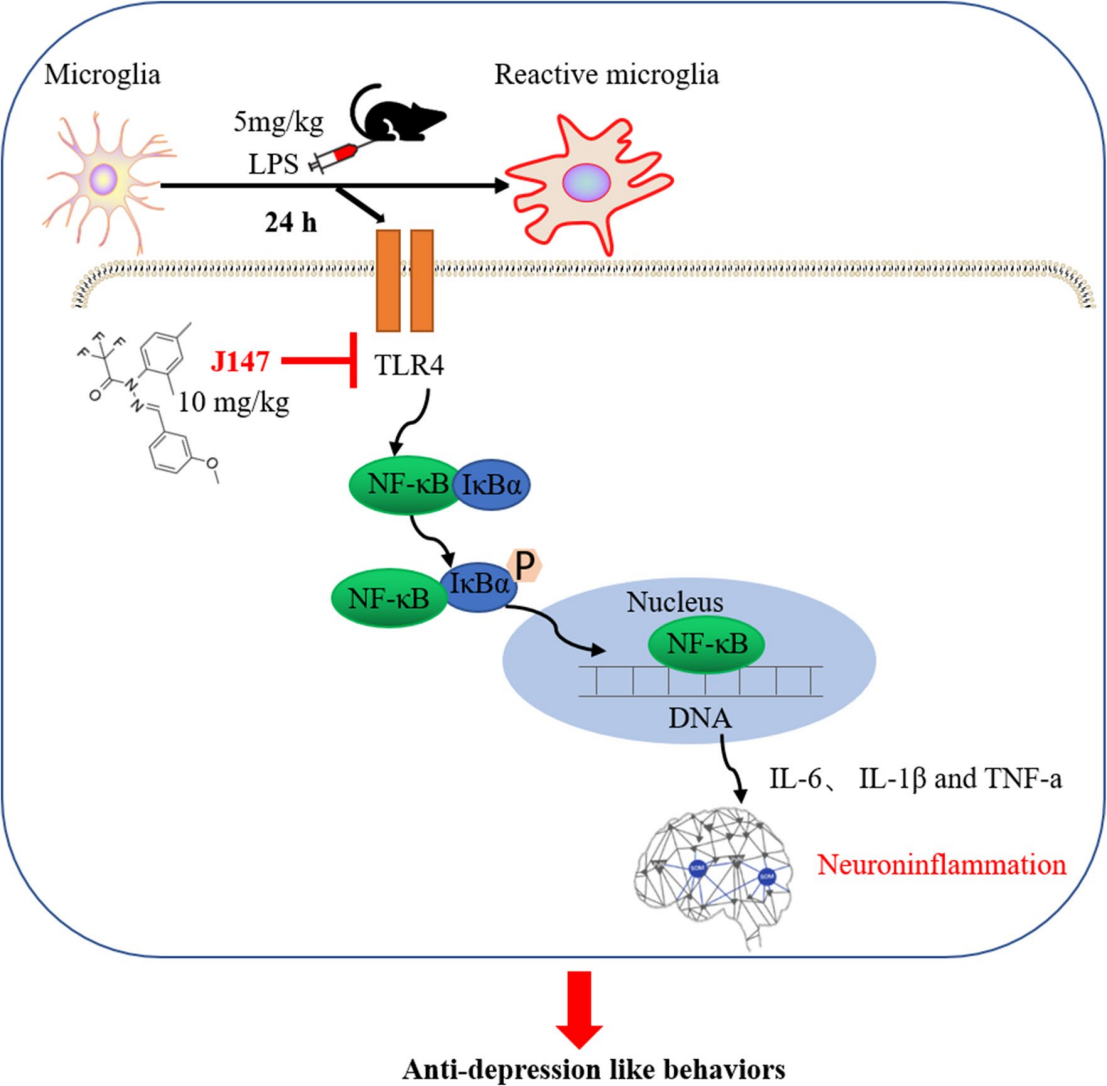
Potential mechanism of anti-depressive effects of J147

Sepsis can affect anyone at any time, but it does tend to occur in very young and old age (Mcpherson et al. 2013), while how it occurs in middle age has been relatively little studied. We established an LPS-induced model of sepsis in middle age mice to mimic the effects of sepsis in humans, and assessed the role of J147 therapy in the depressive-like behaviors observed in SAE. Treatment with J147 could protect mice against bodyweight loss, microglia activation, depressive-like behaviors, and death. J147 administration inhibited the microglia activation-induced inflammatory response in the hippocampus. In addition, J147 suppressed activation of the TLR4/NF-κB signaling pathway in BV2 cells and the hippocampus of mice (Fig. 8). Overall, we elucidated the microglia activation-induced inflammatory response, and noted that the antidepressant effects of J147 in SAE were via the TLR4/NF-κB signaling pathway. These results indicated that J147 could be a candidate drug used to treat SAE.

J147 is a derivative of curcumin and cyclohexyl-bisphenol A (CBA). It is an exciting new compound that is potent, and orally active and prevents Alzheimer’s disease (AD) and diabetic neuropathy (Chen et al. 2011; Daugherty et al. 2018). Studies have shown that J147 administration can improve mitochondrial function, memory, synaptic plasticity, pain, and anxiety and protects neurons against excitotoxicity (Chen et al. 2011; Prior et al. 2013; Daugherty et al. 2018; Goldberg et al. 2018). Herein, we found that pretreatment with J147 significantly reduced the increase in immobility duration in the FST and TST induced by LPS administration, which indicated antidepressant-like effect of J147. These results suggested that J147 treatment ameliorated LPS-induced bodyweight loss, survival, and depressive-like behaviors.

Neuroinflammation has a crucial role in CNS diseases, including AD, Parkinson’s disease (PD), depression, anxiety, sleep disorders, and stroke (Zhu et al. 2012; Mracsko and Veltkamp 2014; Hong et al. 2016; Rossi et al. 2017). Thus, targeting neuroinflammation is a potential strategy to prevent CNS diseases. However, the underlying mechanisms of different CNS diseases are poorly understood. Growing evidence has suggested that neuroinflammation is associated with microglia activation (Muzio et al. 2021). Activated microglia can release proinflammatory cytokines such as IL-6, IL-1β, TNF-α, and ROS (Muzio et al. 2021), thereby leading to neuronal damage. The latter, in turn, induces microglia activation, and then the activated microglia promote further neuronal damage (Block et al. 2007). Thus, inhibiting activated microglia might reduce the neuroinflammatory response. Our data indicated that J147 administration inhibited the LPS-induced inflammatory response in microglia markedly in vitro and in vivo, demonstrating that J147 has an anti-inflammatory effect.

Activation of TLR4 by LPS induces the release of proinflammatory cytokines, which have key roles in the innate immune system. TLR4 has been studied in multiple diseases, and its role in the pathogenesis of inflammatory liver disease has been demonstrated (Soares et al. 2010). Expression of TLR4 and NF-κB has been shown to be increased markedly in the kidneys of LPS-treated rats and LPS-treated NRK-52E cells (Zhang et al. 2017). TLR4 regulates neuronal death (Zhong et al. 2020). Yang and colleagues reported that TLR4 signaling activated NF-κB, which triggered the production of many proinflammtory cytokines, including IL-6, IL-1β, and TNF-α (Yang et al. 2022). Interestingly, the TLR4/NF-κB signaling pathway in the CNS is associated with learning and memory, ischemia, traumatic injury, the proliferation of neural progenitor cell, and the fate of neural progenitor cells (NPC) (Okun et al. 2011). TLR4/NF-κB signaling regulates the activation and polarization of microglia (Xiao et al. 2021b; Zhu et al. 2021). Herein, we explored how J147 inhibited activation of the microglia-induced inflammatory response. We found that J147 administration decreased the LPS-mediated increase in TLR4 expression and nuclear translocation of NF-κB. Hence, the anti-inflammatory effect of J147 was attributable (at least in part) to TLR4-mediated NF-kB inhibition in LPS-stimulated microglia. These results suggested that J147 therapy: (i) blocked IκBα degradation and NF-κB p65 translocation in LPS-treated BV2 cells; (ii) reduced the release of proinflammatory cytokines by regulating the TLR4/NF-κB signaling pathway in BV2 cells.

Our work is not without limitations. First, mitochondrial ATP synthase is a target of J147. The latter modulates aging and dementia through the calmodulin dependent protein kinase 2 (CAMKK2)/AMP activated protein kinase (AMPK)/mTOR pathway (Goldberg et al. 2018). Hence, in LPS-induced systemic inflammation (and even sepsis), whether J147 can regulate mitochondrial function, affect microglial polarization, and activate the CAMKK2/AMPK/mTOR pathway, thereby alleviating organ dysfunction in sepsis, warrants further exploration. Second, we used only male mice a single strain. Gender affects behavioral output, and the role of female mice in LPS-induced behavioral impairment should be explored in the future. Third, we just detected LPS-induced changes only in the hippocampus. Since, other brain regions critical for depression behavior, such as the prefrontal cortex and the amygdala, are importantly affected by LPS, the changes referred above need to also be investigated in these regions.

## Conclusion

Our findings suggest that J147 administration ameliorates LPS-induced neuroinflammation in the hippocampus and exerts antidepressant-like effects by attenuating the TLR4/NF-κB signaling pathway in microglia. We demonstrated that J147, as a powerful neurogenic and neuroprotective drug, could be a candidate for the treatment of sepsis-induced depressive-like behaviors.

## Data Availability

All data generated or analyzed during this study are included in this article. Further inquiries can be directed to the corresponding author.
